# Evaluation of the protective efficacy of six major immunogenic proteins of *Mycoplasma Synoviae*

**DOI:** 10.3389/fvets.2023.1334638

**Published:** 2024-01-04

**Authors:** Shuizhong Han, Ying Wang, Wenchi Chang, Lizhen Wang, Junyang Fang, Jingjing Han, Xiaolan Hou, Xuefeng Qi, Jingyu Wang

**Affiliations:** ^1^College of Veterinary Medicine, Northwest A&F University, Yangling, China; ^2^College of Food and Drugs, Luoyang Polytechnic, Luoyang, China

**Keywords:** *Mycoplasma synoviae*, subunit vaccine, DnaK, enolase, EF-Tu, MSPB, LP78, NADH oxidase

## Abstract

*Mycoplasma synoviae* (*MS*) is a primary avian pathogen prevalent worldwide that causes airsacculitis and synovitis in birds. Vaccination is recommended as the most cost-effective strategy in the control of *MS* infection. Novel alternative vaccines are needed for eradicating and controlling *MS* infection in flocks. DnaK, enolase, elongation factor Tu (EF-Tu), MSPB, NADH oxidase and LP78 are the major immunogenic antigens of *MS* and are promising targets for subunit vaccine candidates. In the present study, genes encoding DnaK, enolase, EF-Tu, MSPB, LP78, and NADH oxidase were cloned and expressed in *Escherichia coli*. Enzyme-linked immunosorbent assay showed that the six recombinant proteins were recognized by convalescent sera, indicating that they were expressed during infection. Two injections of the six subunit vaccines induced a robust antibody response and increased the concentrations of IFN-γ and IL-4, especially rEnolase and rEF-Tu. The proliferation of peripheral blood lymphocytes was enhanced in all of the immunized groups. Chickens immunized with rEnolase, rEF-Tu, rLP78, and rMSPB conferred significant protection against *MS* infection, as indicated by significantly lower DNA copies in the trachea, lower scores of air sac lesions, and lesser tracheal mucosal thickness than that in the challenge control. Especially, rEnolase provided the best protective efficacy, followed by rEF-Tu, rMSPB, and rLP78. Our finds demonstrate that the subunit vaccines and bacterin can only reduce the lesions caused by *MS* infection, but not prevent colonization of the organism. Our findings may contribute to the development of novel vaccine agents against *MS* infection.

## Introduction

*Mycoplasma synoviae* (*MS*) has been described as a major pathogen involved in synovitis, airsacculitis, eggshell apex abnormality, and egg drops (1, 2). *MS* cooperates with other pathogens, such as infectious bronchitis virus (IBV) and Newcastle disease virus (NDV), and tends to exacerbate pathogenesis in chickens (3, 4). Globally, the clinical and economic importance of *MS* has increased since 2000, especially in layer flocks (5).

Generally, *MS* infection can be controlled by three general approaches: maintaining flocks free of infection, medication with antibiotics, and vaccination (6). In China, control and eradication programs for *MS* infection are voluntary; thus, it is difficult to maintain flocks free from *MS*. Although the continuous administration of antibiotics can eventually eradicate *MS* from the farm, the prevalence of multidrug-resistant strains of *MS* increases the risk of reinfection within flocks (7). In this context, reliable vaccines can be a viable option in China. A temperature-sensitive live vaccine MS-H strain has been approved in many countries for use in vaccination and is shown to be safe and efficacious for chickens in the field (8). However, MS-H strain is recommended for *MS*-free birds. Furthermore, the live vaccine can persistently colonize the tracheas of chickens, making *MS* eradication challenging (9). Reportedly, inactivated *MS* can reduce footpad and tracheal lesions in chickens (10). The main disadvantages of bacterins are high cost, the requirement for handling individual birds, and the need for repeated dose to boost avian immune system (6). Hence, vaccines developed shifted to subunit-base vaccines become a viable alternative.

The rational development of effective vaccines is based on the knowledge of antigens involved in protective effects and the host’s immune response to the antigens. The adhesion of mycoplasma to host cells is a prerequisite for colonization and infection (11). Recently, subunit vaccines contain proteins that are involved in *MS* adherence showed effectively protect against *MS* infection (12, 13). In infection caused by *Mycoplasma gallisepticum* (*MG*), another important avian mycoplasma, vaccines containing adherence proteins imparted protective immunity in chickens (14, 15). These findings suggest that the proteins, especially those with adhesion functions, can serve as promising targets for subunit vaccine candidates against *MS* infection. The major immunogenic proteins of *MS* have been identified in previous studies (16–18). Of these, DnaK, enolase, Elongation factor-Tu (EF-Tu), MSPB, and NADH oxidase are involved in mycoplasmas adherence (19–23). Furthermore, the immunological responses and protective effect of DnaK, enolase, EF-Tu and NADH oxidase from other microorganisms have been evaluated. For example, DnaK from *Mycoplasma hyopneumoniae* and *Mycoplasma ovipneumoniae* elicits strong humoral and cellular immune responses (24, 25). In *Mycoplasma suis* and *Streptococcus suis*, the enolase induces a robust immunological response and provides protective efficacy (26, 27). Mice immunized with EF-Tu subunit vaccines are protected from *Streptococcus suis* or *Streptococcus pneumoniae* challenge (28, 29). NADH oxidase functions as a virulence factor in *Mycoplasma hyopneumoniae* and can activate local mucosal immune responses (30). MSPB is the N-terminal part of the variable lipoprotein haemagglutinin (vlhA) that has been identified as a virulence factor of *MS* (31). LP78 is a putative lipoprotein that belongs to the P80 family lipoprotein. Lipoprotein has been identified as a good candidate of antigens for *MS* vaccine (12). The protective efficacy of DnaK, EF-Tu, enolase, MSPB, LP78, and NADH oxidase antigens against *MS* infection has not been reported to date.

The purpose of this study is to evaluate the protective efficacy and immune responses elicited by the six major immunogenic proteins of *MS*, namely DnaK, EF-Tu, enolase, MSPB, LP78, and NADH oxidase. Chickens immunized with purified recombinant proteins conferred significant protection against *MS* infection. The present study may contribute to the development of novel vaccine agents against *MS* infection.

## Materials and methods

### Chicken source and ethics statements

One-day-old Hy-Line Brown hens free of mycoplasma infection were provided by Yangling Lvfang, Co., Ltd. (Shaanxi, China). Feed and water were provided *ad libitum* for the duration of the study. Animal experiments were approved and conducted in accordance with the guidelines of the Ethics Committee in Animal Experimentation of Northwest A&F University (No. 220412).

### Bacterial strains, plasmids, and chicken sera

The *MS* strain W1 was isolated from chickens with several air sac lesions and propagated in modified Frey’s medium at 37°C with 5% CO_2_. *Escherichia coli* (*E. coli*) strains DH5α and BL21 (DE3) were purchased from TransGen Biotech (Beijing, China) and cultured in Luria–Bertani (LB) broth or on solid media. A pET-28a (+) expression vector was obtained from Novagen (Madison, WI, United States). Antisera to *MS* were prepared in chickens by intramuscular inoculation with a commercial inactivated vaccine (YBF-MS1 strain) that was manufactured by Yebio Bioengineering, Co., Ltd. of Qingdao (Qingdao, China). Convalescent sera were obtained from commercial poultry farms with known mycoplasma infection status. The sera were all ensured by the commercial *MS* antibody test kit (IDEXX, Westbrook, Maine, United States).

### Construction of six recombinant plasmids containing genes encoding rDnaK, rEnolase, rEF-Tu, rMSPB, rLP78, and rNADH

The features of DnaK, enolase, EF-Tu, MSPB, LP78, and NADH oxidase of *MS* are shown in Table 1. The conservation of *dnak*, *enolase*, *ef-tu*, *mspb*, *lp78*, and *nadh* genes was investigated by multiple-sequence alignments with reference sequences. The signal peptide sequences were predicted using SignaIP-5.0 server.^1^ For the cloning and site-directed mutagenesis of the genes, several pairs of primers were designed based on the complete *MS* strain MS-H genome sequence in the GenBank database (accession number: CP021129) and synthesized by Tsingke Biotechnology Co., Ltd. (Beijing, China). The primer sequences are summarized in Supplementary Table S1. The genomic DNA of *MS* strain MS-H (Bioproperties Ltd., Australia) was extracted using the TIANamp Bacteria DNA Kit (Tiangen, Beijing, China). PCR was performed in a final volume of 50 μL (2 μL of each primer, 25 μL of 2 × PrimeSTAR Max Premix, 19 μL of ultrapure water, and 2 μL of template DNA). The PCR conditions were as follows: 94°C for 10 min; 35 cycles of 95°C for 30 s, 50–60°C for 30 s, 72°C for 60 s, and 72°C for 10 min. Sequences that contained tryptophan codons (TGA) were subjected to site-directed mutagenesis to TGG using overlap PCR. The mutagenesis was further confirmed by nucleotide sequencing by Tsingke Biotechnology Co., Ltd. (Beijing, China). The amplified products were ligated into the pET-28a (+) vector and transformed into *E. coli* DH5α and BL21 (DE3) competent cells by the heat shock method.

**Table 1:** Features of the selected genes with numbers of amino acids and TGA codons.

No	Protein names	Gene names	Amino (aa)	Molecular Mass (kDa)	TGA codons
1	Chaperone protein DnaK	MSH_01775	596	69	2
2	Enolase	MSH_00070	452	53	2
3	Elongation factor Tu	MSH_03475	394	44	0
4	MSPB	MSH_01355	313	38	2
5	LP78	MSH_01690	770	86	6
6	NADH oxidase	MSH_02670	458	50	3

### Expression, purification, and identification of rDnaK, rEnolase, rEF-Tu, rMSPB, rLP78, and rNADH

*E. coli* BL21 (DE3) cells containing the recombinant plasmids were cultured in LB broth supplemented with kanamycin (50 μg/mL) at 37°C on a shaker at 200 rpm. In the mid-log phase, *E. coli* BL21 (DE3) cells were treated with 1 mM IPTG for 12 h at a temperature ranging from 20 to 25°C. The cells were harvested and washed twice with Tris–HCl (0.02 mol/L, pH 8.0), and sonicated on ice with 5-s pulses at 15-s intervals. After centrifugation, the supernatant containing the recombinant protein was applied to an affinity chromatography column prepacked with Ni-NTA His-Bind^®^ Resin (Huiyan bio, Wuhan, China). The recombinant protein was eluted using a linear gradient of 20–500 mM of imidazole. The concentrations of recombinant proteins were measured using a BCA Protein Assay kit (Beyotime, China) according to the manufacturer’s instructions.

### Reactivity of the six recombinant proteins to *MS*-positive sera from chicken

Enzyme-linked immunosorbent assay (ELISA) was performed to assess the reactivity of recombinant proteins with convalescent sera and sera from chickens immunized with the *MS* bacterin vaccine. Briefly, polystyrene microtiter ELISA plates were coated with 1 μg/mL of recombinant proteins or the whole *MS* cells diluted in sodium carbonate buffer (pH 9.6) and incubated overnight at 4°C. After washing three times with phosphate buffer saline (PBS) supplemented with 0.05% Tween-20 (PBST), the plates were blocked with 5% skim milk in PBST for 2 h at 37°C. After three washes, 1:500 dilution serum samples were added to each well and incubated at 37°C for 2 h. After three more washes, 1:10,000 dilution HRP-conjugated rabbit anti-chicken antibody (ABclonal, Hubei, China) was added to each well, and the plates were incubated at 37°C for 1 h. After three more washes, 3,3′,5,5′-tetramethylbenzidine substrate solution (Beyotime, China) was added, and the colorimetric reaction was developed for 15 min at 37°C. The reaction was terminated by adding 2 mol/L H_2_SO_4_. Finally, the optical density (OD) values were measured at 450 nm.

### Preparation of vaccines containing the six recombinant proteins and immunization of chickens

**Figure 1:**
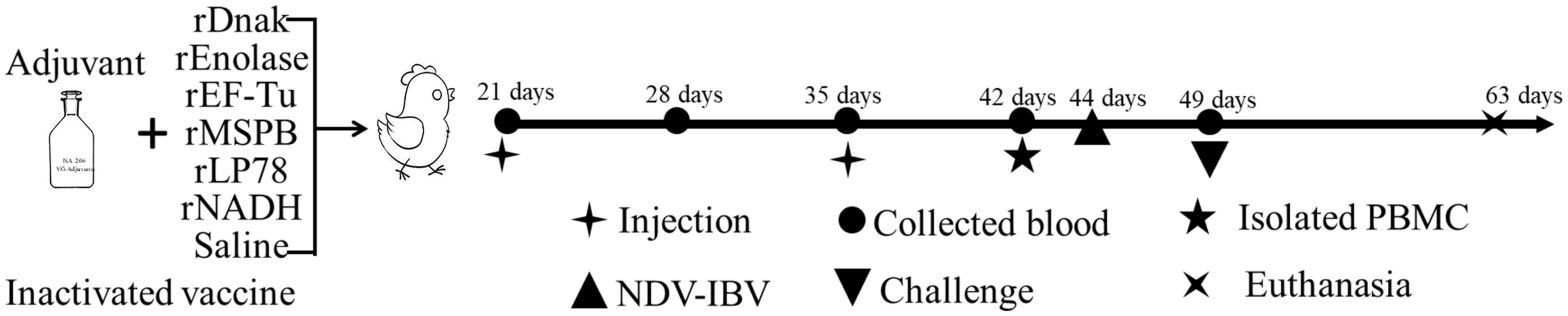
Schematic diagram of the immunization and challenge experiment in chickens. One hundred chickens were randomly divided into 10 groups (*n* = 10 per group) and immunized subcutaneously twice with subunit vaccines. A commercial inactivated MS vaccine and saline emulsified with adjuvant were set as controls.

To evaluate the immune effects of the prepared recombinant proteins, the proteins (diluted to 200 μg/mL) and saline were emulsified with Montanide^™^ ISA 206 VG adjuvant (Seppic, Shanghai, China) at an equal ratio (w/w) individually. One hundred chickens were randomly divided into 10 groups (*n* = 10 per group), including group 1, rDnaK; group 2, rEnolase; group 3, rEF-Tu; group 4, rMSPB; group 5, rLP78; group 6, rNADH; group 7, inactivated vaccine; group 8, challenge control; group 9, NDV/IBV vaccine control; and group 10, Frey’s broth control. At 21 days of age, each chicken received 0.5 mL of subunit vaccine (groups 1 to group 6), inactivated vaccine (YBF-MS1 strain) (group 7) or saline (saline + adjuvant) (groups 8 to group 10) injected subcutaneously into the nape of the neck. A booster injection was administered after 14 days. The experimental design is shown in Figure 1.

### Antibodies responses against the recombinant proteins by ELISA

Blood samples were collected weekly after immunization, and the prepared sera were stored at −80°C before processing. Antibodies responses generated by immunization with subunit and inactivated vaccines were determined by ELISA using recombinant proteins or whole *MS* individually. The coating concentrations and serum dilutions were determined through checkerboard titration. ELISA was conducted as described above and the optical density (OD) values were measured at 450 nm.

### Detection of cytokine secretion in sera by ELISA

At 14 and 21 days after the first immunization, the interferon-gamma (IFN-γ) and interleukin-4 (IL-4) levels in the sera were measured. Assays were performed using commercial chicken IFN-γ and IL-4 ELISA kits (Wuhan Saipei Biotechnology Co., Ltd., China) according to the manufacturer’s instructions. Briefly, blank wells, standard wells, and sample wells were arranged. After dilution, 50 μL of the sample was added to the coating plate along with 50 μL of biotin-labeled anti-IFN-γ/IL-4 antibody, and the plate was incubated at 37°C for 1 h. After three washes, 50 μL of HRP-labeled streptavidin was added, and the plate was incubated at 37°C for 30 min. After three more washes, 100 μL of the chromogenic solution was added to each well, and the plate was incubated at 37°C for 10 min. The reaction was terminated by adding 50 μL of terminator solution to each well. The optical density value (OD) of the sample was measured at 450 nm. The corresponding concentration of the sample was calculated by the standard curve, plotting the OD_450nm_ values against different concentrations of the standard sample.

### Lymphocyte proliferation assay

Chicken peripheral blood lymphocytes were isolated at 21 days post the first immunization using a commercial kit (Solarbio, Beijing, China). After three washes with PBS, the lymphocytes were suspended at 10^6^ cells/mL in RPMI1640 medium (HyClone, America) supplemented with 10% FBS (HyClone, America) and seeded in 96-well flat-bottomed plates in triplicate at 100 μL per well. The cells were then stimulated with 100 μL of purified recombinant proteins (50 μg/mL) or culture medium (negative control) at 37°C for 48 h. At the end of incubation, 10 μL of 3-(4,5-dimethyl-2-thiazolyl)-2,5-diphenyl-2-H- tetrazolium bromide (MTT) (Beyotime, China) was added, and the cells were cultured for 4 h. Then, 100 μL of formazan was added, and the cells were incubated for 4 h until the crystals dissolved. The absorbance value of each well was measured at 570 nm. Stimulation index (SI) values were calculated as the ratios of the optical density (OD) values of antigen-stimulated wells to those of unstimulated ones.

### Challenging chickens with *MS*

Four weeks post the first immunization, chickens in groups 1 to 8 were challenged by the intratracheal route with 100 μL of *MS* W1 strain culture (1 × 10^8^ CCU/mL). Groups 9 and 10 received equal volume of modified Frey’s medium. To increase the incidence and severity of mycoplasma lesions, chickens in groups 1–9 were administered one dose of a commercial Newcastle disease and infectious bronchitis vaccine (Strain La sota + Strain LDT3-A) (Harvac Biotechnology Co., Ltd., Harbin, China) by intratracheal route 5 days before the challenge and group 10 received equal volume of saline.

### Isolation and quantitative real-time PCR (qRT–PCR) of *MS* in tracheal swabs from chickens

Swabs were collected from the tracheas of chickens in all groups at 14 days after challenge and then inoculated on modified Frey’s agar and cultured at 37°C for up to 2 weeks. To quantitate *MS* in swabs, total DNA was extracted from the swabs using a TIANamp Bacteria DNA Kit (Tiangen, Beijing, China). The 16S-23S rDNA ISR fragment (217 bp) of *MS* was amplified by a pair of primers, as described previously (32). The primers sequences were as follows, MS-F: GAGAAGCAAAATAGTGAT ATC, MS-R: CAGTCGTCTCCGAAGTTAACAA. The qRT–PCR conditions were as follows: each PCR mixture of 25 μL contained 0.5 μL of each primer (10 pmol), 12.5 μL of 2× TransStart^®^Top/Tip Green qPCR SuperMix, 9.5 μL of ultrapure water, and 2 μL of template DNA. The qRT–PCR program was as follows: 94°C for 10 min; 40 cycles of 94°C for 5 s, 60°C for 30 s. Sterile water and positive plasmid were used for the negative and positive controls, respectively. The *MS* DNA copies were calculated according to the standard curve plotting the Ct values against 10-fold serial dilutions of the standard plasmid.

### Air sac lesions and tracheal mucosal pathology score evaluation

At 14 days post the challenge, chickens were euthanized and necropsied. Gross lesions of the air sacs were visually assessed and scored on a scale of 0 to 4, as described by Kleven et al. (4). The trachea of each bird was fixed in 4% neutral buffered formalin, dehydrated, embedded in paraffin, solidified, cut, and stained with hematoxylin and eosin. The stained tracheal tissues were examined microscopically, and the thickness of the mucosa was measured. The mean mucosal thickness of each bird was calculated and then averaged in each group.

### Statistical analysis

Significant differences in the lymphocyte proliferation assay, cytokine concentrations, mean DNA copies numbers, air sac lesion scores, and mean tracheal mucosal thickness were analyzed with one-way analysis of variance (ANOVA) using IBM SPSS Statistics 20. A *p* value of 0.05 was considered significant. Graphs were prepared in GraphPad Prism 8.0.

## Results

### Expression, purification, and identification of rDnaK, rEnolase, rEF-Tu, rMSPB, rLP78, and rNADH

**Figure 2:**
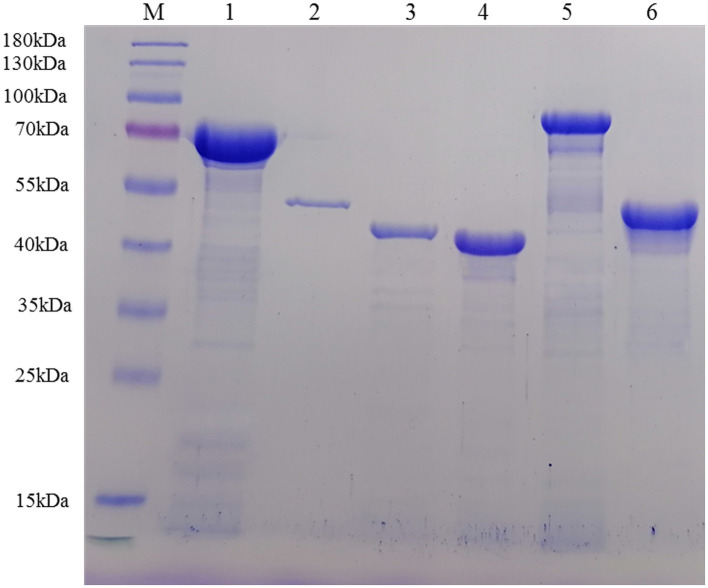
Recombinant proteins were purified by Ni^+^ affinity chromatography and analyzed by SDS–PAGE. Lane M: protein marker; Lane 1: rDnaK; Lane 2: rEnolase; Lane 3: rEF-Tu; Lane 4: rMSPB; Lane 5: rLP78; Lane 6: rNADH oxidase.

Multiple sequence alignments revealed that *dnak*, *enolase*, *ef-tu*, *lp78*, and *nadh* genes shared ≥90% identities with the references sequences, while *mspb* was 84.5–90.2% to the references sequences (Supplementary Table S2). Sequence analysis indicated that TGA codons in the selected genes were successfully mutated into TGG. Sequences without TGA codons and signal peptide sequences were ligated into the pET-28a (+) vector successfully and then used to transform *E. coli* BL21 (DE3) cells. After induction, the recombinant proteins were primarily found in the supernatant of bacterial cell lysates (Supplementary Figures S1–S6). The recombinant proteins containing 6 × His-tag at the N-terminus were successfully purified by a Ni^+^ affinity chromatography column (Figure 2) and could be recognized by anti-His monoclonal antibodies (Supplementary Figure S7). After dialysis, the concentrations of the purified recombinant proteins ranged from 0.2 to 2.0 mg/mL.

### Evaluation of the reactivity of six recombinant proteins with *MS*-positive sera from chickens

**Figure 3:**
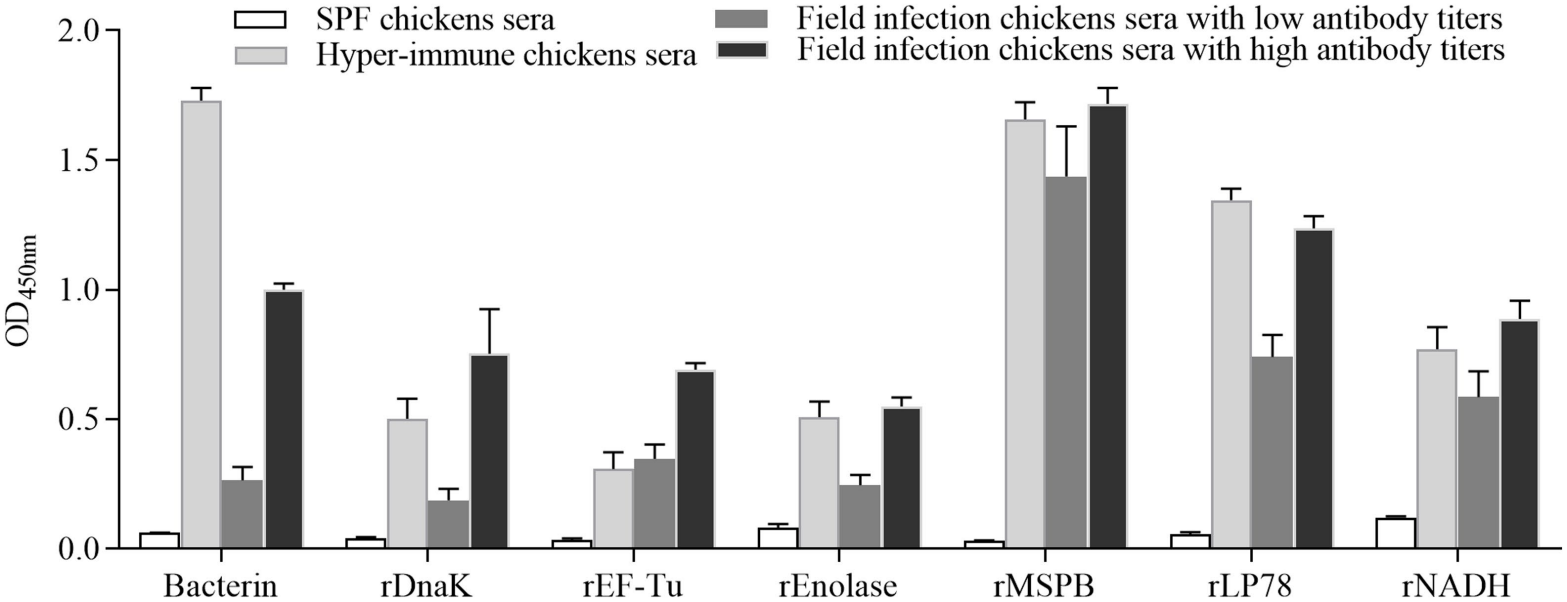
Reactions among recombinant proteins with *MS* convalescent sera and immune sera. The data represent the analyses of sera from 5 chickens in each group. Bars represent the mean ± standard deviation of the absorbance at 450 nm in each group.

To evaluate the reactivity of six recombinant proteins, indirect ELISA was performed using convalescent sera and sera from chickens immunized with *MS* bacterin. The serum levels of antibodies against *MS* were measured using *MS* strain W1. The six recombinant proteins showed recognition by convalescent sera and immune sera against *MS* (Figure 3). Notably, stronger binding was observed on rMSPB, followed by that on rLP78.

### Determination of antibody responses in chickens using ELISA

**Figure 4:**
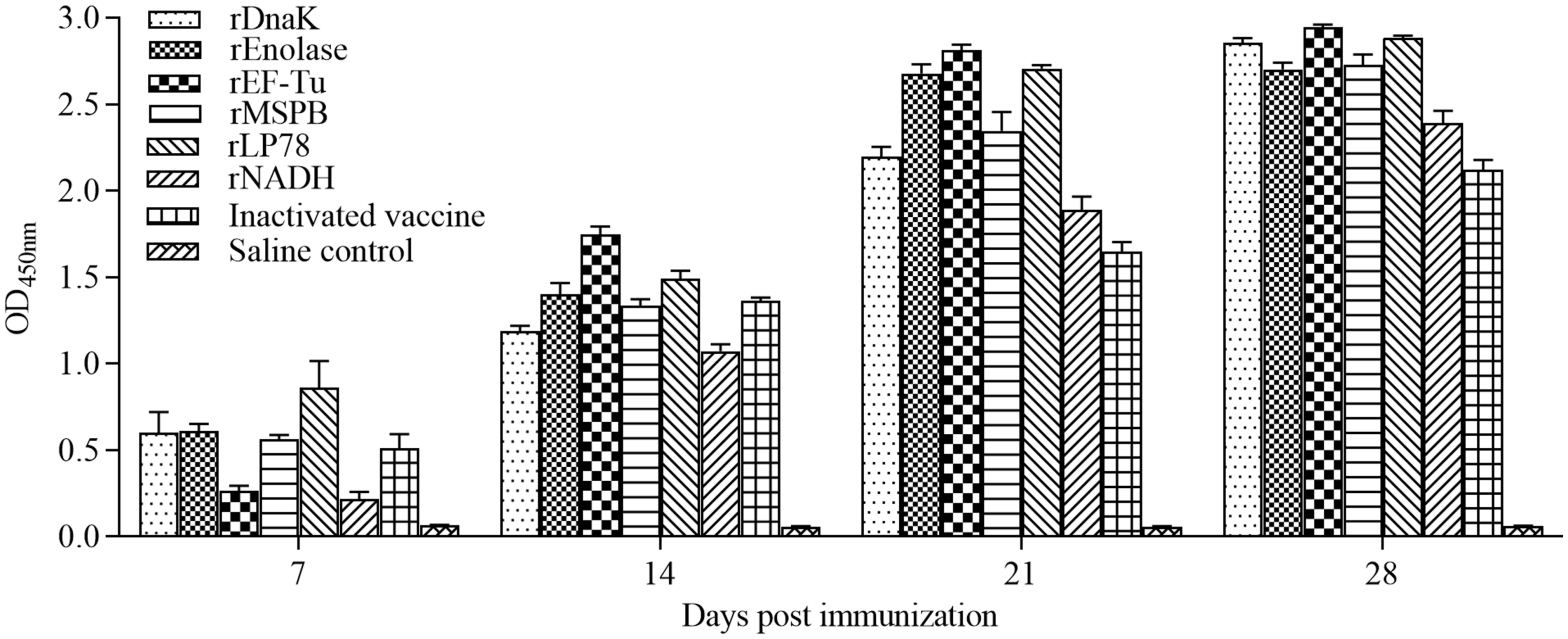
Antibodies levels in sera of the immunized chickens. Chickens (*n* = 10) were immunized twice with a two-week interval using the subunit vaccines, inactivated vaccine, and saline. Antibodies against the recombinant proteins or whole *MS* were detected by ELISA at 7, 14, 21, and 28 days after the first immunization. The serum samples were tested using rDnaK (100 ng/well) at a dilution of 1:400, rEnolase (20 ng/well) at a dilution of 1:200, rEF-Tu (100 ng/well) at a dilution of 1:600, rMSPB (20 ng/well) at a dilution of 1:400, rLP78 (100 ng/well) at a dilution of 1:600, and rNADH (20 ng/well) at a dilution of 1:400. Bars represent the mean ± standard deviation of the absorbance at 450 nm in each group.

To evaluate humoral immune responses, sera were collected weekly after the first immunization and detected by indirect ELISA. Chickens vaccinated with the subunit vaccines or inactivated vaccine became seropositive at 7 days after the first immunization (Figure 4). The optical density of the antibodies at 450 nm (OD_450nm_) increased gradually. In the rEnolase, rEF-Tu, and rLP78 groups, the OD_450nm_ peaked at 21 days, whereas it peaked at 28 days in the other groups. Chickens in the saline control groups showed negative results during the immunization stage.

### Measurement of IFN-γ and IL-4 secretion in sera

**Figure 5:**
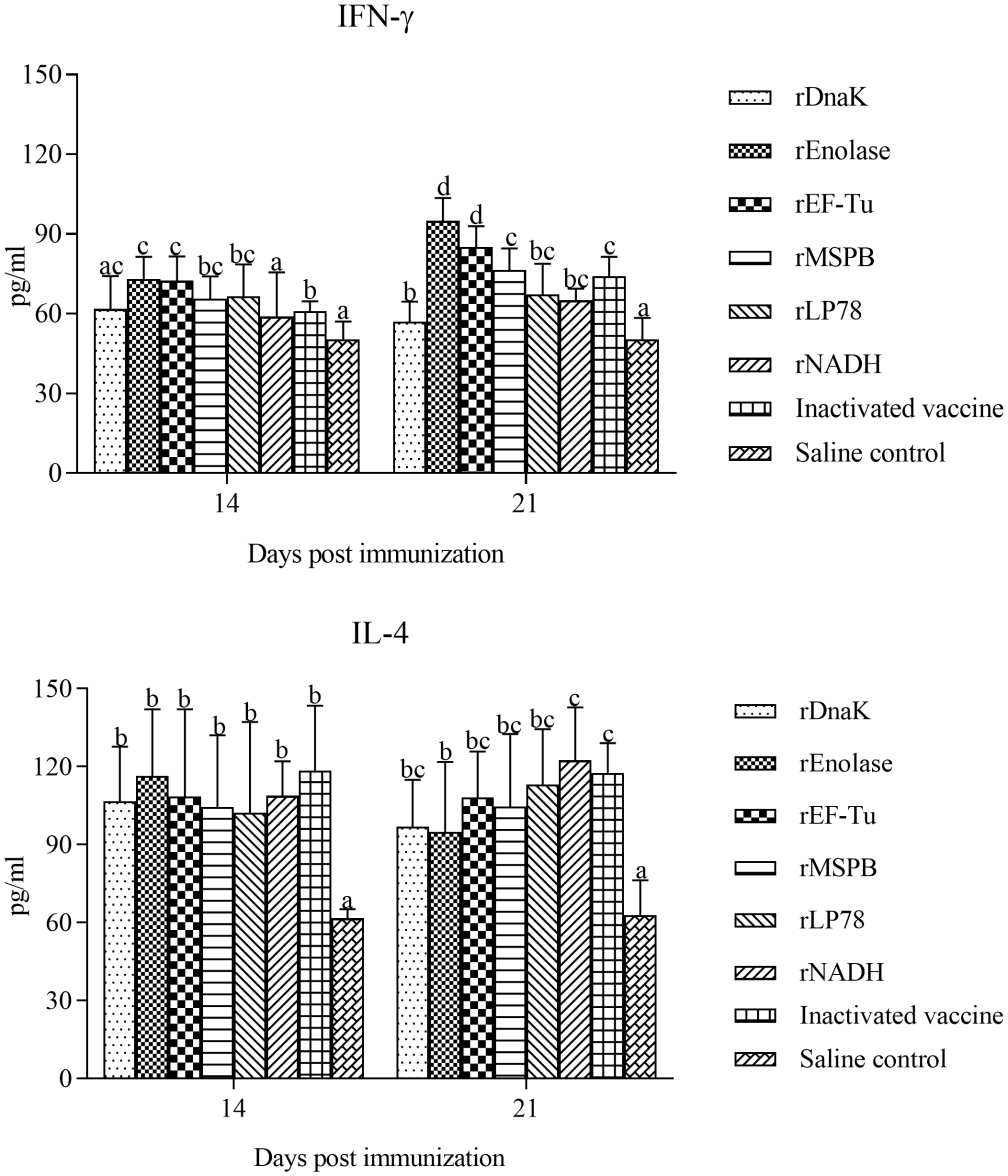
Vaccination induces the levels of IFN-γ and IL-4 in chicken serum. The serum samples from the subunit vaccine-, inactivated vaccine- and saline-immunized chickens were analyzed for the cytokines IFN-γ and IL-4 by ELISA. Serum samples were collected at 14 and 21 days post the first immunization. Bars represent the mean ± standard deviation of the concentrations of IFN-γ and IL-4 in each group. The differences were compared between each vaccinated group. The same letter indicates no obvious difference (*p >* 0.05); different letters indicate a significant difference (*p <* 0.05).

The levels of IFN-γ and IL-4 in the sera of the immunized chickens were determined at 14 and 21 days post the first immunization using an ELISA kit. Compared with the saline control group, the subunit vaccine and inactivated vaccine groups showed a remarkable (*p <* 0.05) increase in the levels of IFN-γ and IL-4 (Figure 5). In particular, the rEnolase and rEF-Tu groups exhibited significantly (*p <* 0.05) higher levels of IFN-γ than the inactivated vaccine group at 21 days. Barring that in the rEnolase group, no obvious difference (*p >* 0.05) in the IL-4 levels was observed among the subunit vaccine and inactivated vaccine groups.

### Lymphocyte proliferation assay

**Figure 6:**
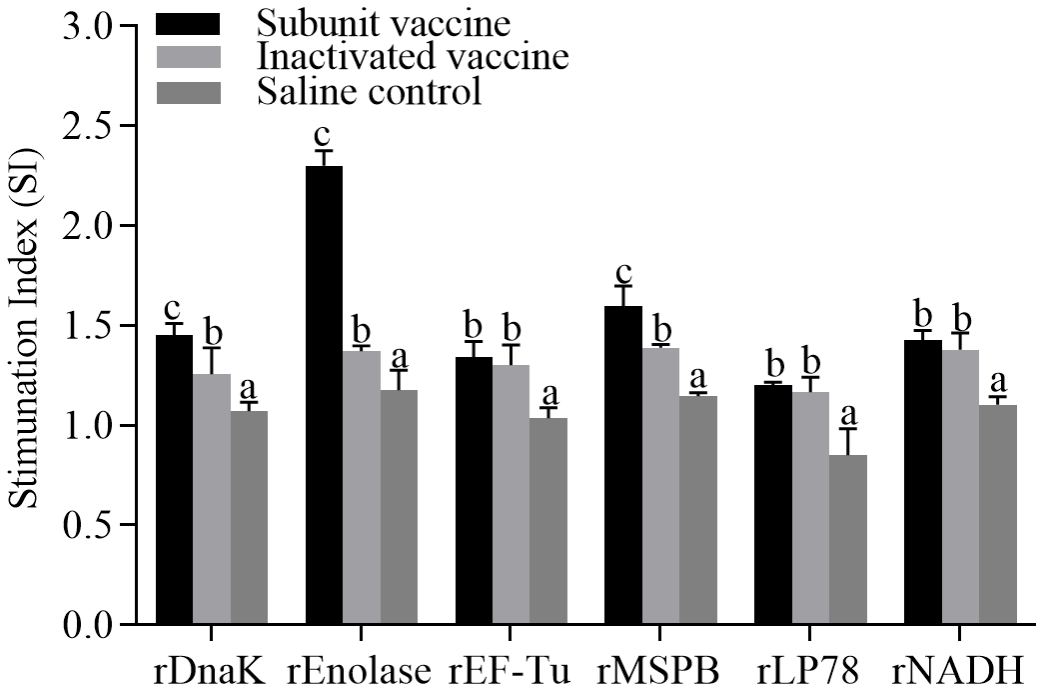
Lymphocyte proliferative assays from chickens vaccinated with recombinant subunit vaccines and inactivated vaccine. The PBMCs were isolated at 21 days post the first immunization. Recombinant proteins were administered to stimulate PBMCs. The stimulation index (SI) values were compared between the vaccinated groups and the saline control. The same letter indicates no obvious difference (*p >* 0.05); different letters indicate significant differences (*p <* 0.05).

To determine the effect of the recombinant proteins on the cellular immune responses, chicken peripheral blood lymphocytes were isolated at 21 days post the first immunization and stimulated with the recombinant proteins, respectively. The lymphocyte proliferative responses were measured and the SI values were shown in Figure 6. The levels of SI in the subunit vaccine and inactivated vaccine groups were significantly (*p <* 0.05) higher than those in the saline control group. Compared with those in the inactivated vaccine group, the levels of SI in the rDnaK, rEnolase, and rMSPB groups were significantly (*p <* 0.05) higher.

### Isolation and qRT–PCR of *MS* samples from the trachea of chicken

**Figure 7:**
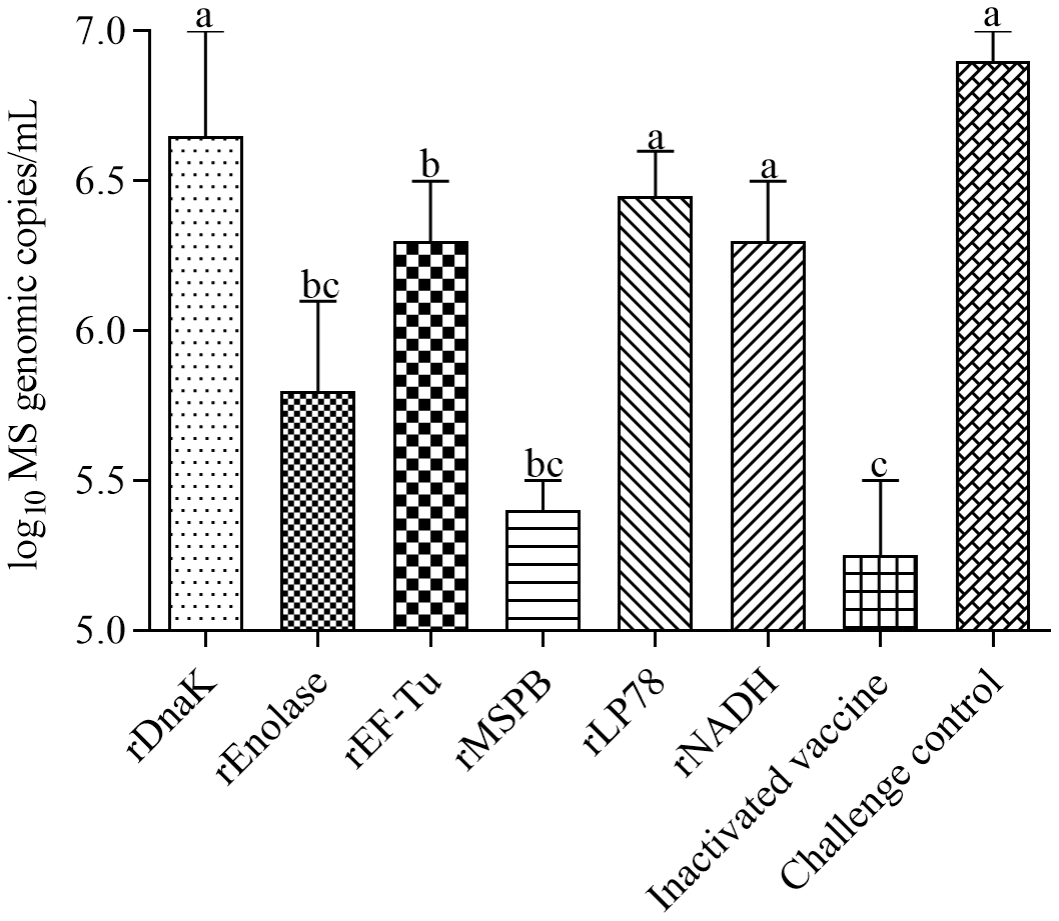
Mean values of the number of *MS* genomic copies in tracheal swabs. Tracheal swabs were collected at 14 days after challenge. Variation is expressed as the standard deviation. The same letter indicates no obvious difference (*p* > 0.05); different letters indicate significant differences (*p* < 0.05).

The incidence of *MS* isolation and the number of *MS* DNA copies in tracheal swabs were measured at 14 days post the challenge. *MS* was isolated from nearly all chickens in groups 1 to 8 after challenge. No *MS* DNA copies were detected in the NDV-IBV control (group 9) and saline control (group 10). As shown in Figure 7, chickens in the rEnolase, rEF-Tu and rMSPB groups had significantly (*p <* 0.05) lower *MS* DNA copies in swabs compared to the challenge control group (group 8). No significant difference (*p >* 0.05) was observed among the rEnolase, rMSPB and inactivated vaccines immunized groups.

### Evaluation of air sac lesions and tracheal mucosal pathology score

**Figure 8:**
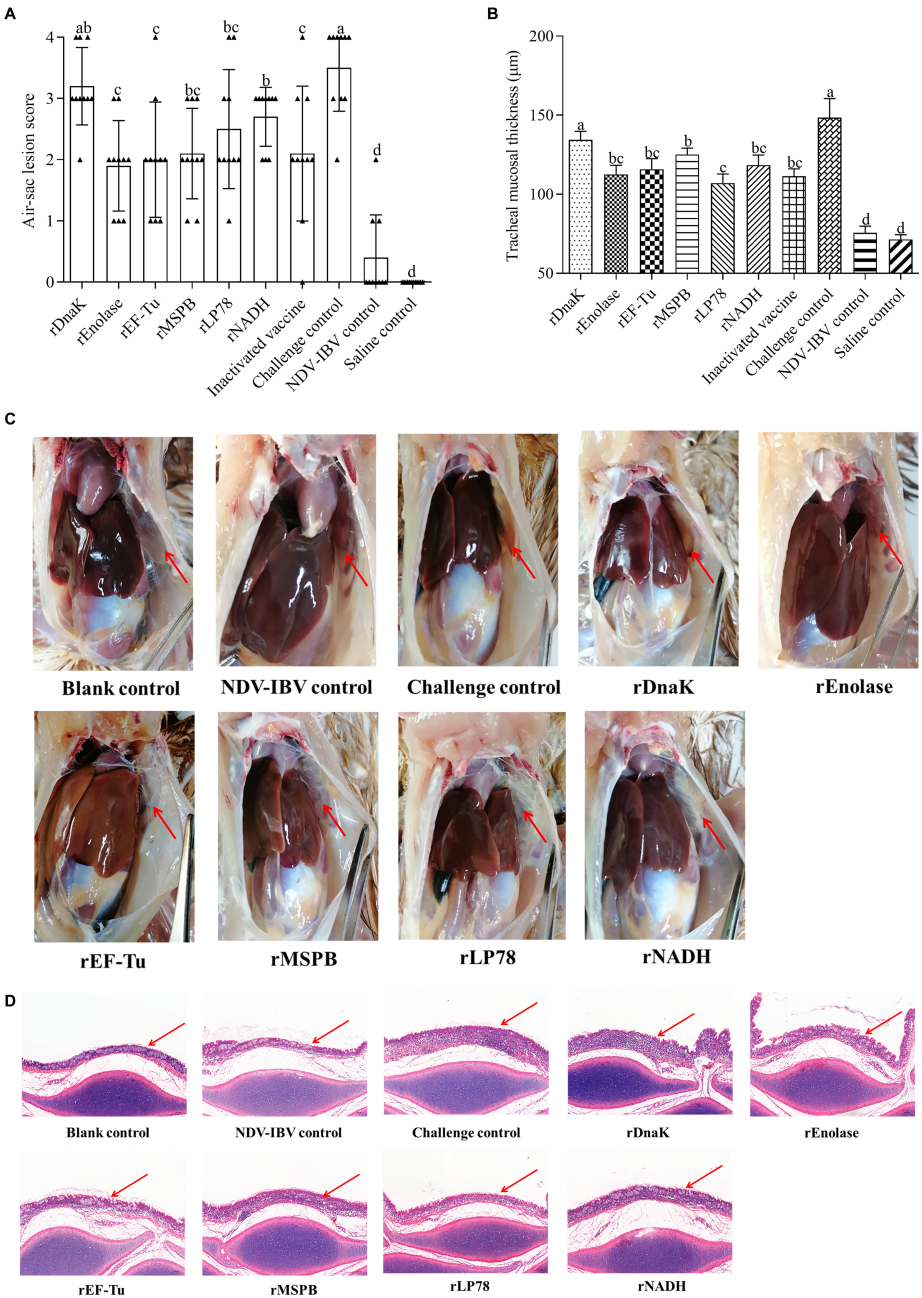
Gross air sac lesions and tracheal mucosa thickness. Chickens were necropsied at 14 days post the challenge by exposure to *MS* strain W1 and NDV-IBV vaccine. Air sac lesions and tracheal mucosa thickness were measured and averaged in each group. **(A)** Air sac lesion scores in each chicken was visually assessed and scored on a scale of 0 to 4. **(B)** Mean tracheal mucosa thickness in each group. **(C)** Macroscopic air sac lesion in chickens at 14 days post challenge. **(D)** Trachea was stained with hematoxylin and eosin (100×). Variation is expressed as the standard deviation. The same letter indicates no obvious difference (*p* > 0.05); different letters indicate significant differences (*p* < 0.05). The red arrow represents the anterior aic sac or tracheal mucosa.

The total scores of air sac lesions and the mean thickness of the tracheal mucosa were measured at 14 days post challenge. As shown in Figures 8A,C, chickens in the challenge control group showed severe airsacculitis, whereas the NDV-IBV vaccine control and the saline control groups had slight or no airsacculitis. Except for those in the rDnaK group, chickens in the immunized groups had significantly fewer (*p <* 0.05) air sac lesions than those in the challenge control group. In particular, no significant (*p >* 0.05) difference was observed in the scores of air sac lesions among the rEnolase, rEF-Tu, rMSPB, rLP78, and inactivated vaccine groups. Except for that in the rDnaK group, significantly (*p <* 0.05) less mucosal thicknesses were observed in the immunized groups compared with that in the challenged groups (Figures 8B,D). No significant difference (*p >* 0.05) was observed in the mucosal thicknesses between the rEnolase, rEF-Tu, rLP78, rNADH, and inactivated vaccine groups.

## Discussion

*MS* infection is one of the most cost-intensive diseases in the poultry industry (1, 2). Vaccination plays an important role in the prevention of *MS* infection (6). However, the currently live and inactivated vaccines have some disadvantages. Therefore, new alternative vaccines need to be developed, which are more efficacious and less expensive. Nevertheless, limited data are available on the protective effect of *MS* antigens. DnaK, enolase, Elongation factor-Tu (EF-Tu), MSPB, and NADH oxidase, and LP78 are the major immunogenic proteins of *MS* (18). DnaK is one of the most conserved proteins and belongs to the heat shock protein (HSP) 70 family of molecular chaperones (23). enolase is one of the best characterized glycolytic enzyme (20). Elongation factor-Tu (EF-Tu) is one of the most abundant and conserved bacterial proteins and plays a key role in protein synthesis (33). MSPB is the N-terminal part of vlhA that is responsible for the attachment of *MS* to chicken erythrocytes (34). MSPB and LP78 are major *MS* lipoproteins (18, 31). NADH oxidase belongs to the largest group of enzyme oxidoreductases, which functions in catalyzing the oxidation of NAD^+^ to NADH by simultaneously reducing of O_2_ to H_2_O or H_2_O_2_ (21). Additionally, DnaK, enolase, Elongation factor-Tu (EF-Tu), MSPB and NADH oxidase are identified to be located on the membrane of mycoplasmas (20, 21, 23, 35, 36).

The immune mechanisms by which *MS* vaccines provide protection are complex and not yet well understood. Bursectomized chickens demonstrate more severe airsacculitis and synovitis than normal chickens, which suggests that humoral immune responses play an important role in resistance to *MS* infection (37, 38). In the present study, the six recombinant proteins were recognized by the sera from chickens immunized or infected with *MS*. In addition, a robust humoral immune response was induced by the six subunit vaccines. These finds indicate that the six proteins stimulate the humoral immune responses and are expressed during infection. Compared with the challenge group, chickens immunized with the subunit vaccines (except for rDnaK) showed significantly lower numbers of *MS* DNA copies in the tracheal swabs, lower scores of airsacculitis and less tracheal mucosal thicknesses. Adhesion to host cells is a prerequisite for mycoplasma infection and a precondition for successful colonization (11). In previous studies, antibodies against recombinant DnaK, enolase, EF-Tu, MSPB and NADH can inhibit mycoplasmas adherence and growth (20, 21, 23, 34, 35). Thus, the humoral immune responses are assumed to contribute to the protective immunity.

Although the numbers of *MS* DNA copies in the tracheal swabs, scores of airsacculitis and thickness of the tracheal mucosa were significantly lower in chickens inoculated with the subunit vaccines, the protective efficacy were still insufficient than anticipated. A multitude of factors could contribute to insufficient protection efficacy. First of all, these may be explained by the fact that mycoplasmas cytadherence is a complex, multifactorial process involving numerous membrane proteins and cytoskeletal elements (11). In addition to the antigens used in the present study, other proteins have also been proven to contribute to the adhesion process of *MS* (12). Secondly, the limited protective effect on the colonization of *MS* in the trachea may be attributed to the lack of specific antibodies in the trachea, which is consistent with earlier findings in *MG*. Reportedly, the levels of antibodies in the respiratory tract play an important role against the colonization of *MG*, and the predominant Ig class in respiratory secretions is IgG (39, 40). Although the *MG* bacterins stimulate a strong humoral immune response, low levels of specific IgG in tracheobronchial washes are detected (41, 42). In *Mycoplasma hyopneumoniae*, the bacterins provide only partial protection and do not prevent colonization of the organism (43, 44). Thirdly, the divergent antigenic variations can protect *MS* against elimination by the host’s immune system. vlhA is the most abundant and important virulence factor of *MS*, but it undergoes phase variable expression, and highly divergent antigenic variants are generated owing to pseudogene insertions (34, 45). Additionally, the inoculum of NDV-IBV vaccine priors to *MS* exposure exacerbates the pathogenicity of *MS* infection (3, 4). Finally, chickens were inoculated with large dose of *MS*, which was considerably greater than that in natural infection.

Cytokines can induce and amplify inflammatory and immune responses by recruiting and activating cells, which mediate crucial functions in host defenses against bacterial or viral infections (46). IL-4 is known to play important roles in Th2 type humoral immune responses, and IFN-γ is an important cytokine involved in Th1 type cellular immune responses (47). Compared with the saline control, the six subunit vaccines induced a significantly increase in IL-4 levels. These data are consistent with the results of the high levels of antibodies against the recombinant proteins. This suggests that the six subunit vaccines can induce robust humoral immune responses. Previous studies have shown that *MS* exerts considerable mitogenic effects on lymphocytes and can stimulate lymphocytes to produce IFN-γ (10, 48, 49). In MS-H inoculated chickens, a significantly increase of IFN-γ is observed in the tracheal mucosa, and the local cellular response is dominated by a Th-17 response (50). In the present study, the levels of IFN-γ were significantly upregulated (*p* < 0.05) in chickens inoculated with the subunit vaccines. Especially, the rEnolase and rEF-Tu subunit vaccines groups showed significantly higher IFN-γ levels than the other groups. These data indicate the six subunit vaccines induce cellular immune responses, while the rEnolase and rEF-Tu groups produced the best immune effect. In addition, a lower score of air sac lesions was observed in the rEnolase and rEF-Tu groups. IFN-γ cooperates with IL-17 (predominantly produced by Th17-type cells), activating macrophages to kill phagocytic pathogens and infected cells (47). In other species of mycoplasmas, Dnak, EF-Tu, and enolase demonstrated increased levels of IFN-γ and could induce more IgG1 and IgG2a antibodies, which may have led to a mixed Th1/Th2-type immune response (25, 26, 51, 52). Whether the six subunit vaccines induced a mixed Th1/Th2-type immune response should be further determined. Additionally, the proliferation of lymphocytes induced by the six recombinant proteins was analyzed. The SI values of the subunit vaccine groups were marginally higher than those of the saline control group, while rEnolase subunit vaccine produced the best immune effect. This indicates the recombinant proteins induced T-cell responses. The mechanism of protection induced by immunization with the subunit vaccines remains incompletely defined. However, the findings of this study suggest that both humoral and cellular immune responses contribute to the protective efficacy of subunit vaccines against *MS* infection.

## Conclusion

In conclusion, rDnaK, rEF-Tu, rEnolase, rMSPB, rLP78, and rNADH can stimulate both humoral and cellular immune responses in chickens. Barring rDnaK, the subunit vaccines and bacterin significantly reduce the lesions caused by *MS* infection, but do not prevent colonization of the organism. These findings may contribute to the development of novel vaccines against *MS*.

## Data availability statement

The original contributions presented in the study are included in the article/Supplementary material, further inquiries can be directed to the corresponding author.

## Ethics statement

The animal study was approved by Ethics Committee in Animal Experimentation of Northwest A&F University. The study was conducted in accordance with the local legislation and institutional requirements.

## Author contributions

SH: Data curation, Formal analysis, Investigation, Software, Validation, Writing – original draft. YW: Data curation, Formal analysis, Software, Writing – review & editing. WC: Data curation, Formal analysis, Writing – review & editing. LW: Methodology, Supervision, Writing – review & editing. JF: Data curation, Writing – review & editing. JH: Data curation, Writing – review & editing. XH: Data curation, Writing – review & editing. XQ: Methodology, Writing – review & editing. JW: Conceptualization, Funding acquisition, Methodology, Project administration, Resources, Writing – review & editing.

## Footnotes

^1^https://services.healthtech.dtu.dk/services/SignalP-5.0/

## References

[1] Kleven SH Fletcher OJ Davis RB. Influence of strain of *Mycoplasma synoviae* and route of infection on development of synovitis or airsacculitis in broilers. Avian Dis. (1975) 19:126–35. doi: 10.2307/1588963, PMID: 1120029

[2] Cisneros-Tamayo M Kempf I Coton J Michel V Bougeard S de Boisseson C. Investigation on eggshell apex abnormality (EAA) syndrome in France. Isolation of *Mycoplasma synoviae* is frequently associated with *Mycoplasma pullorum*. BMC Vet Res. (2020) 16:271. doi: 10.1186/s12917-020-02487-0 32758240 PMC7404918

[3] Hopkins SR Yoder HW Jr. Influence of infectious bronchitis strains and vaccines on the incidence of *Mycoplasma synoviae* airsacculitis. Avian Dis. (1982) 26:741–52. doi: 10.2307/1589860, PMID: 6297445

[4] Kleven SH King DD Anderson DP. Airsacculitis in broilers from *Mycoplasma synoviae*. Effect on air-sac lesions of vaccinating with infectious bronchitis and Newcastle virus. Avian Dis. (1972) 16:915–24. doi: 10.2307/1588772, PMID: 5079882

[5] Landman WJ. Is *Mycoplasma synoviae* outrunning *Mycoplasma gallisepticum*? A viewpoint from the Netherlands. Avian Pathol. (2014) 43:2–8. doi: 10.1080/03079457.2014.881049, PMID: 24397240

[6] Kleven SH. Control of avian mycoplasma infections in commercial poultry. Avian Dis. (2008) 52:367–74. doi: 10.1637/8323-041808-Review.1, PMID: 18939621

[7] Zhang X Guo M Xie D Chen Y Zhang C Cao Y. Antibiotic resistance of *Mycoplasma synoviae* strains isolated in China from 2016 to 2019. BMC Vet Res. (2022) 18:1. doi: 10.1186/s12917-021-03104-4 34980113 PMC8722301

[8] Markham JF Scott PC Whithear KG. Field evaluation of the safety and efficacy of a temperature-sensitive *Mycoplasma synoviae* live vaccine. Avian Dis. (1998) 42:682–9. doi: 10.2307/1592703, PMID: 9876836

[9] Moronato ML Cecchinato M Facchetti G Mainenti M Gobbo F Catania S. Application of different laboratory techniques to monitor the behaviour of a *Mycoplasma synoviae* vaccine (MS-H) in broiler breeders. BMC Vet Res. (2018) 14:357. doi: 10.1186/s12917-018-1669-8 30458824 PMC6245925

[10] Gong X Chen Q Ferguson-Noel N Stipkovits L Szathmary S Liu Y. Evaluation of protective efficacy of inactivated *Mycoplasma synoviae* vaccine with different adjuvants. Vet Immunol Immunopathol. (2020) 220:109995. doi: 10.1016/j.vetimm.2019.109995, PMID: 31877484

[11] Razin S Jacobs E. *Mycoplasma* adhesion. J Gen Microbiol. (1992) 138:407–22. doi: 10.1099/00221287-138-3-407, PMID: 1593256

[12] Zhang G Han L Li Z Chen Y Li Q Wang S. Screening of immunogenic proteins and evaluation of vaccine candidates against *Mycoplasma synoviae*. NPJ Vaccines. (2023) 8:121. doi: 10.1038/s41541-023-00721-y 37582795 PMC10427712

[13] Zhang G Han L Zhao Y Li Q Wang S Shi H. Development and evaluation of a multi-epitope subunit vaccine against *Mycoplasma synoviae* infection. Int J Biol Macromol. (2023) 253:126685. doi: 10.1016/j.ijbiomac.2023.127597, PMID: 37666406

[14] Zhang D Long Y Li M Gong J Li X Lin J. Development and evaluation of novel recombinant adenovirus-based vaccine candidates for infectious bronchitis virus and *Mycoplasma gallisepticum* in chickens. Avian Pathol. (2018) 47:213–22. doi: 10.1080/03079457.2017.1403009, PMID: 29115156

[15] Saito S Fujisawa A Ohkawa S Nishimura N Abe T Kodama K. Cloning and DNA sequence of a 29 kilodalton polypeptide gene of *Mycoplasma gallisepticum* as a possible protective antigen. Vaccine. (1993) 11:1061–6. doi: 10.1016/0264-410X(93)90134-J, PMID: 8212828

[16] Noormohammadi AH Markham PF Markham JF Whithear KG Browning GF. *Mycoplasma synoviae* surface protein MSPB as a recombinant antigen in an indirect ELISA. Microbiology (Reading). (1999) 145:2087–94. doi: 10.1099/13500872-145-8-2087, PMID: 10463175

[17] Gurevich VA Ley DH Markham JF Whithear KG Walker ID. Identification of *Mycoplasma synoviae* immunogenic surface proteins and their potential use as antigens in the enzyme-linked immunosorbent assay. Avian Dis. (1995) 39:465–74. doi: 10.2307/1591797, PMID: 8561729

[18] Bercic RL Slavec B Lavric M Narat M Bidovec A Dovc P. Identification of major immunogenic proteins of *Mycoplasma synoviae* isolates. Vet Microbiol. (2008) 127:147–54. doi: 10.1016/j.vetmic.2007.07.020, PMID: 17720337

[19] Bencina D Narat M Dovc P Drobnic-Valic M Habe F Kleven SH. The characterization of *Mycoplasma synoviae* EF-Tu protein and proteins involved in hemadherence and their N-terminal amino acid sequences. FEMS Microbiol Lett. (1999) 173:85–94. doi: 10.1111/j.1574-6968.1999.tb13488.x, PMID: 10220885

[20] Bao S Guo X Yu S Ding J Tan L Zhang F. *Mycoplasma synoviae* enolase is a plasminogen/fibronectin binding protein. BMC Vet Res. (2014) 10:223. doi: 10.1186/s12917-014-0223-6 25253294 PMC4189797

[21] Hu Z Li H Zhao Y Wang G Shang Y Chen Y. NADH oxidase of *Mycoplasma synoviae* is a potential diagnostic antigen, plasminogen/fibronectin binding protein and a putative adhesin. BMC Vet Res. (2022) 18:455. doi: 10.1186/s12917-022-03556-2 36581820 PMC9798693

[22] Bencina D. Haemagglutinins of pathogenic avian mycoplasmas. Avian Pathol. (2002) 31:535–47. doi: 10.1080/0307945021000024526, PMID: 12593736

[23] Li Y Wang J Liu B Yu Y Yuan T Wei Y. DnaK functions as a moonlighting protein on the surface of *Mycoplasma hyorhinis* cells. Front Microbiol. (2022) 13:842058. doi: 10.3389/fmicb.2022.842058, PMID: 35308339 PMC8927758

[24] Jorge S de Oliveira NR Marchioro SB Fisch A Gomes CK Hartleben CP. The *Mycoplasma hyopneumoniae* recombinant heat shock protein P42 induces an immune response in pigs under field conditions. Comp Immunol Microbiol Infect Dis. (2014) 37:229–36. doi: 10.1016/j.cimid.2014.07.001, PMID: 25082621

[25] Jiang F He J Navarro-Alvarez N Xu J Li X Li P. Elongation factor Tu and heat shock protein 70 are membrane-associated proteins from *Mycoplasma ovipneumoniae* capable of inducing strong immune response in mice. PLoS One. (2016) 11:e0161170. doi: 10.1371/journal.pone.0161170, PMID: 27537186 PMC4990256

[26] Xue S Seo K Yang M Cui C Yang M Xiang S. *Mycoplasma suis* alpha-enolase subunit vaccine induces an immune response in experimental animals. Vaccines (Basel). (2021) 9:1506. doi: 10.3390/vaccines9121506, PMID: 34960252 PMC8708218

[27] Feng Y Pan X Sun W Wang C Zhang H Li X. *Streptococcus suis* enolase functions as a protective antigen displayed on the bacterial cell surface. J Infect Dis. (2009) 200:1583–92. doi: 10.1086/644602, PMID: 19848587

[28] Feng L Niu X Mei W Li W Liu Y Willias SP. Immunogenicity and protective capacity of EF-Tu and FtsZ of *Streptococcus suis* serotype 2 against lethal infection. Vaccine. (2018) 36:2581–8. doi: 10.1016/j.vaccine.2018.03.079, PMID: 29627237

[29] Nagai K Domon H Maekawa T Hiyoshi T Tamura H Yonezawa D. Immunization with pneumococcal elongation factor Tu enhances serotype-independent protection against *Streptococcus pneumoniae* infection. Vaccine. (2019) 37:160–8. doi: 10.1016/j.vaccine.2018.11.015, PMID: 30442480

[30] Xu L Hao F Wang J Feng Z Zhang L Yuan T. Th1 and Th17 mucosal immune responses elicited by nasally inoculation in mice with virulence factors of *Mycoplasma hyopneumoniae*. Microb Pathog. (2022) 172:105779. doi: 10.1016/j.micpath.2022.105779, PMID: 36116609

[31] Narat M Bencina D Kleven SH Habe F. The hemagglutination-positive phenotype of *Mycoplasma synoviae* induces experimental infectious synovitis in chickens more frequently than does the hemagglutination-negative phenotype. Infect Immun. (1998) 66:6004–9. doi: 10.1128/IAI.66.12.6004-6009.1998, PMID: 9826385 PMC108761

[32] Raviv Z Kleven SH. The development of diagnostic real-time TaqMan PCRs for the four pathogenic avian mycoplasmas. Avian Dis. (2009) 53:103–7. doi: 10.1637/8469-091508-Reg.1, PMID: 19432011

[33] Voorhees RM Ramakrishnan V. Structural basis of the translational elongation cycle. Annu Rev Biochem. (2013) 82:203–36. doi: 10.1146/annurev-biochem-113009-092313, PMID: 23746255

[34] Khiari AB Mardassi BB. Characterization of the antigenic and functional domains of a *Mycoplasma synoviae* variant vlhA gene. Vet Microbiol. (2012) 156:322–9. doi: 10.1016/j.vetmic.2011.11.016, PMID: 22176762

[35] Yu Y Wang H Wang J Feng Z Wu M Liu B. Elongation factor thermo unstable (EF-Tu) moonlights as an adhesin on the surface of *Mycoplasma hyopneumoniae* by binding to fibronectin. Front Microbiol. (2018) 9:974. doi: 10.3389/fmicb.2018.00974 29867877 PMC5962738

[36] Noormohammadi AH Markham PF Whithear KG Walker ID Gurevich VA Ley DH. *Mycoplasma synoviae* has two distinct phase-variable major membrane antigens, one of which is a putative hemagglutinin. Infect Immun. (1997) 65:2542–7. doi: 10.1128/iai.65.7.2542-2547.1997, PMID: 9199417 PMC175359

[37] Vardaman TH Landreth K Whatley S Dreesen LJ Glick B. Resistance to *Mycoplasma synoviae* is bursal dependent. Infect Immun. (1973) 8:674–6. doi: 10.1128/iai.8.4.674-676.1973, PMID: 4200544 PMC422909

[38] Kume K Kawakubo Y Morita C Hayatsu E Yoshioka M. Experimentally induced synovitis of chickens with *Mycoplasma synoviae*. Effects of bursectomy and thymectomy on course of the infection for the first four weeks. Am J Vet Res. (1977) 38:1595–600. PMID: 931142

[39] Avakian AP Ley DH. Protective immune response to *Mycoplasma gallisepticum* demonstrated in respiratory-tract washings from *M. gallisepticum*-infected chickens. Avian Dis. (1993) 37:697–705. doi: 10.2307/1592017, PMID: 8257359

[40] Yagihashi T Tajima M. Antibody responses in sera and respiratory secretions from chickens infected with *Mycoplasma gallisepticum*. Avian Dis. (1986) 30:543–50. doi: 10.2307/1590419, PMID: 3767815

[41] Talkington FD Kleven SH. Evaluation of protection against colonization of the chicken trachea following administration of *Mycoplasma gallisepticum* bacterin. Avian Dis. (1985) 29:998–1003. doi: 10.2307/1590452, PMID: 3833239

[42] Elfaki MG Kleven SH Jin LH Ragland WL. Sequential intracoelomic and intrabursal immunization of chickens with inactivated *Mycoplasma gallisepticum* bacterin and iota carrageenan adjuvant. Vaccine. (1992) 10:655–62. doi: 10.1016/0264-410X(92)90085-X, PMID: 1523875

[43] Ahn Y Yang S Oh T Park KH Cho H Suh J. Efficacy evaluation of a bivalent vaccine containing porcine circovirus type 2b and *Mycoplasma hyopneumoniae* against an experimental dual challenge. Front Vet Sci. (2021) 8:652313. doi: 10.3389/fvets.2021.652313, PMID: 33996979 PMC8119751

[44] Yang S Oh T Park KH Cho H Chae C. A dual swine challenge with porcine circovirus type 2 (PCV2) and *Mycoplasma hyopneumoniae* used to compare a combination of mixable monovalent PCV2 and monovalent *M. hyopneumoniae* vaccines with a ready-to use PCV2 and *M. hyopneumoniae* bivalent vaccine. Front Vet Sci. (2020) 7:579. doi: 10.3389/fvets.2020.00579 32984414 PMC7492382

[45] Noormohammadi AH Markham PF Duffy MF Whithear KG Browning GF. Multigene families encoding the major hemagglutinins in phylogenetically distinct mycoplasmas. Infect Immun. (1998) 66:3470–5. doi: 10.1128/IAI.66.7.3470-3475.1998, PMID: 9632627 PMC108374

[46] Toews GB. Cytokines and the lung. Eur Respir J Suppl. (2001) 34:3s–17s. doi: 10.1183/09031936.01.00266001, PMID: 12392030

[47] Yin D He L Zhu E Fang T Yue J Wen M. A fowl adenovirus serotype 4 (FAdV-4) Fiber2 subunit vaccine candidate provides complete protection against challenge with virulent FAdV-4 strain in chickens. Vet Microbiol. (2021) 263:109250. doi: 10.1016/j.vetmic.2021.109250, PMID: 34649009

[48] Cole BC Aldridge KE Ward JR. Mycoplasma-dependent activation of normal lymphocytes. Mitogenic potential of mycoplasmas for mouse lymphocytes. Infect Immun. (1977) 18:393–9. doi: 10.1128/iai.18.2.393-399.1977, PMID: 924676 PMC421245

[49] Cole BC Overall JC Jr Lombardi PS Glasgow LA. Induction of interferon in ovine and human lymphocyte cultures by mycoplasmas. Infect Immun. (1976) 14:88–94. doi: 10.1128/iai.14.1.88-94.1976, PMID: 985808 PMC420848

[50] Omotainse OS Wawegama NK Kulappu Arachchige SN Coppo MJ Vaz PK Woodward AP. Tracheal cellular immune response in chickens inoculated with *Mycoplasma synoviae* vaccine, MS-H or its parent strain 86079/7NS. Vet Immunol Immunopathol. (2022) 251:110472. doi: 10.1016/j.vetimm.2022.110472, PMID: 35940079

[51] Galli V Simionatto S Marchioro SB Fisch A Gomes CK Conceição FR. Immunisation of mice with *Mycoplasma hyopneumoniae* antigens P37, P42, P46 and P95 delivered as recombinant subunit or DNA vaccines. Vaccine. (2012) 31:135–40. doi: 10.1016/j.vaccine.2012.10.088, PMID: 23137841

[52] Chen YL Wang SN Yang WJ Chen YJ Lin HH Shiuan D. Expression and immunogenicity of *Mycoplasma hyopneumoniae* heat shock protein antigen P42 by DNA vaccination. Infect Immun. (2003) 71:1155–60. doi: 10.1128/IAI.71.3.1155-1160.2003, PMID: 12595427 PMC148838

