# Supplementary material for: Evaluation of the protective efficacy of six major immunogenic proteins of Mycoplasma Synoviae
Source: Front Vet Sci. 2024 Jan 4;10:1334638. doi: 10.3389/fvets.2023.1334638 (PMC10794622; doi:10.3389/fvets.2023.1334638)
Supplement: Supplementary file 1 [file Data_Sheet_1.docx]

Supplementary Material

## Supplementary Figures


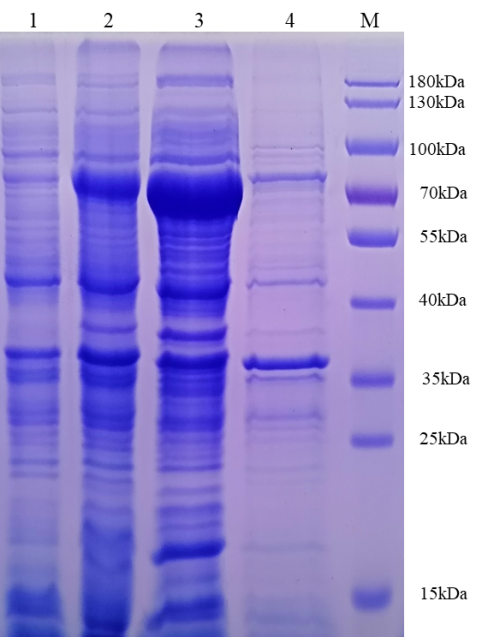


**Figure S1.** Solubility analysis of recombinant DnaK expression in *E. coli*.Lane M: protein marker; Lane 1: Whole cell lysates of *E. coli* BL21 (pET-28a-dnak) before IPTG induction; Lane 2: Whole cell lysates of *E. coli* BL21 (pET-28a-dnak) after IPTG induction; Lane 3: Supernatants from IPTG-induced *E. coli* BL21 (pET-28a-dnak); 4: Sediments from IPTG-induced *E. coli* BL21 (pET-28a-dnak).


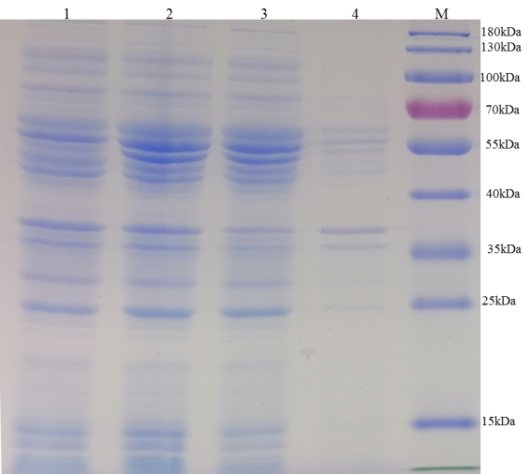


**Figure S2.** Solubility analysis of recombinant Enolase expression in *E. coli*. Lane M: protein marker; Lane 1: Whole cell lysates of *E. coli* BL21 (pET-28a-enolase) before IPTG induction; Lane 2: Whole cell lysates of *E. coli* BL21 (pET-28a- enolase) after IPTG induction; Lane 3: Supernatants from IPTG-induced *E. coli* BL21 (pET-28a- enolase); 4: Sediments from IPTG-induced *E. coli* BL21 (pET-28a- enolase).


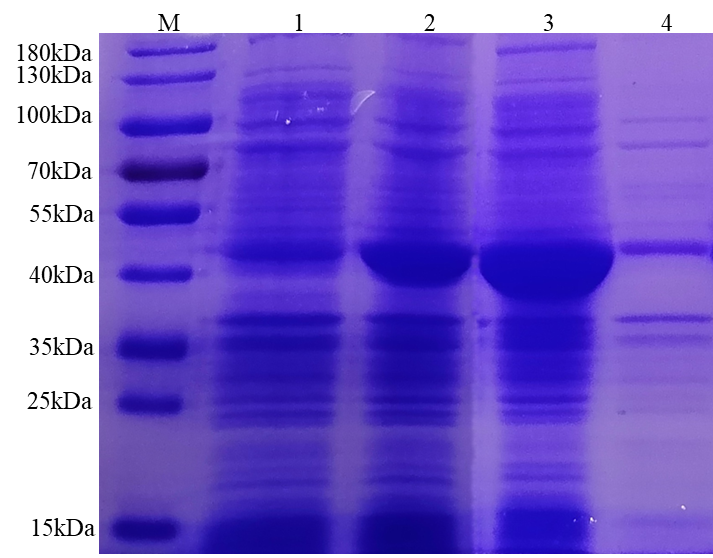


**Figure S3.** Solubility analysis of recombinant Ef-tu expression in *E. coli*. Lane M: protein marker; Lane 1: Whole cell lysates of *E. coli* BL21 (pET-28a-ef-tu) before IPTG induction; Lane 2: Whole cell lysates of *E. coli* BL21 (pET-28a-ef-tu) after IPTG induction; Lane 3: Supernatants from IPTG-induced *E. coli* BL21 (pET-28a-ef-tu); 4: Sediments from IPTG-induced *E. coli* BL21 (pET-28a-ef-tu).


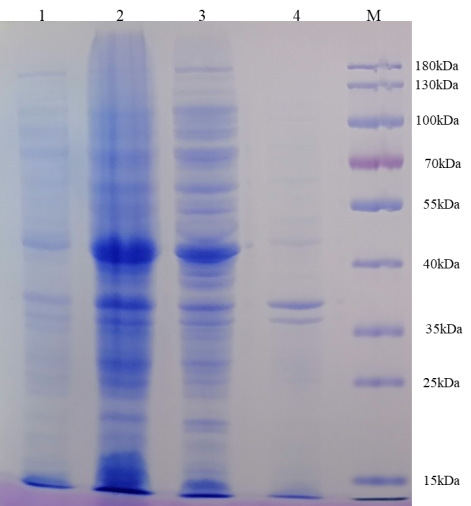


**Figure S4.** Solubility analysis of recombinant MSPB expression in *E. coli*. Lane M: protein marker; Lane 1: Whole cell lysates of *E. coli* BL21 (pET-28a-mspb) before IPTG induction; Lane 2: Whole cell lysates of *E. coli* BL21 (pET-28a-mspb) after IPTG induction; Lane 3: Supernatants from IPTG-induced *E. coli* BL21 (pET-28a-mspb); Lane 4: Sediments from IPTG-induced *E. coli* BL21 (pET-28a-mspb).


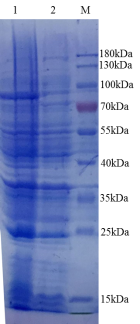

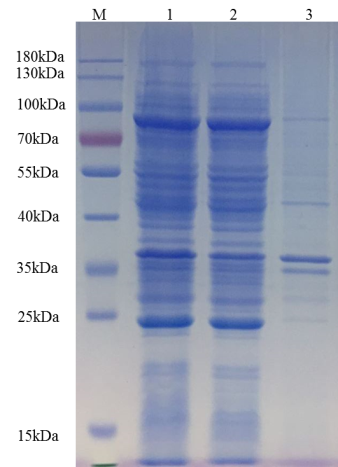


A B

**Figure S5.** Solubility analysis of recombinant LP78 expression in *E. coli*. A: Lane M: protein marker; Lane 1: Whole cell lysates of *E. coli* BL21 (pET-28a-lp78) before IPTG induction; Lane 2: Whole cell lysates of *E. coli* BL21 (pET-28a- lp78) after IPTG induction. B: Lane M: protein marker; Lane 1: Whole cell lysates of *E. coli* BL21 (pET-28a- lp78) after IPTG induction. Lane 2: Supernatants from IPTG-induced *E. coli* BL21 (pET-28a- lp78); 3: Sediments from IPTG-induced *E. coli* BL21 (pET-28a- lp78).


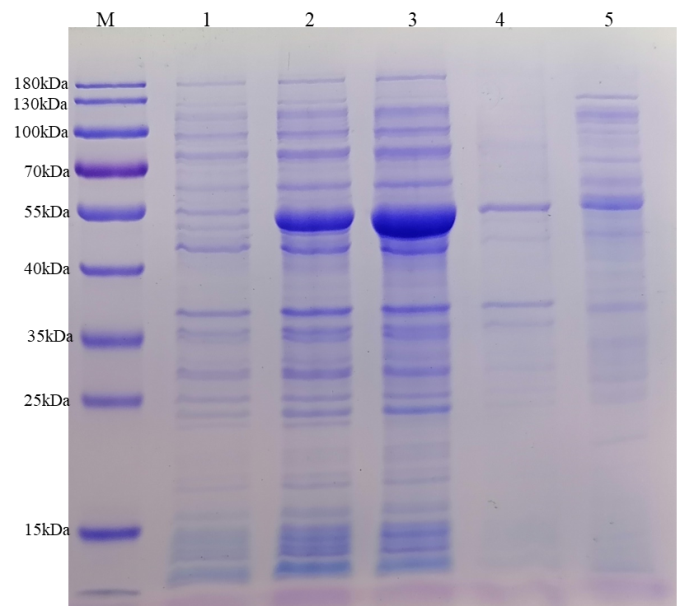


**Figure S6.** Solubility analysis of recombinant NADH expression in *E. coli*. Lane M: protein marker; Lane 1: Whole cell lysates of *E. coli* BL21 (pET-28a-nox) before IPTG induction; Lane 2: Whole cell lysates of *E. coli* BL21 (pET-28a- nox) after IPTG induction; Lane 3: Supernatants from IPTG-induced *E. coli* BL21 (pET-28a- nox); 4: Sediments from IPTG-induced *E. coli* BL21 (pET-28a- nox).


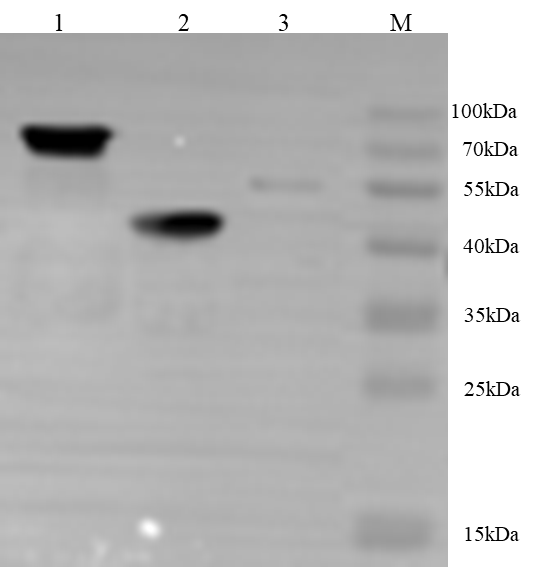

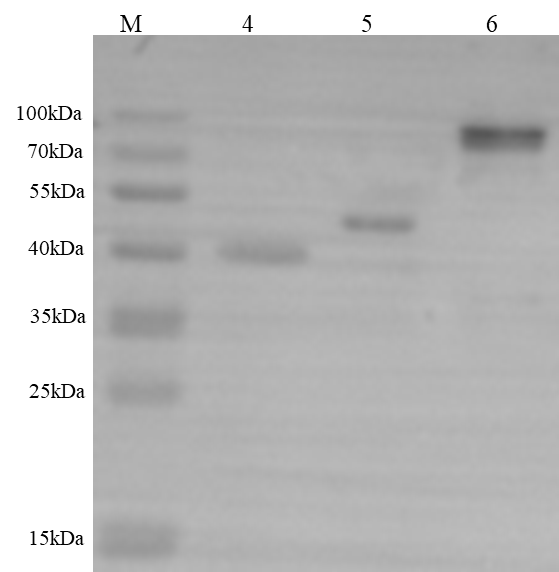


A B

**Figure S7.** Identification of the recombinant proteins by Western blotting using mouse anti-His monoclonal antibody as the primary antibody. A: Lane M: protein marker; Lane 1: rDnaK; Lane 2: rEnolase; Lane 3: rEf-Tu; B: Lane 4: rMSPB; Lane 5: rLP78; Lane 6: rNADH oxidase.

**Supplementary Table**

**Table S1.** Primers used for overlap PCR amplification of the selected genes.

| **No.** | **Protein names** | **Gene names** | **Primer names** | **Sequence 5’-3’** | **Length** | **Amplicon size (bp)** |
| --- | --- | --- | --- | --- | --- | --- |
| 1 | Chaperone protein DnaK | MSH_01775 | dnaK-1F | ATGGCAAAAGAAATAGTATTAGG | 23 | 635 |
|  |  |  | dnaK-1R | GTTAACCATTTAACTATTTCGTTATCCCAATCATCTC | 30 |  |
|  |  |  | dnaK-2F | GAGATGATTGGGATAACGAAATAGTTAAATGGTTAAC | 35 | 1190 |
|  |  |  | dnaK-2R | TTCTTCTACAACTGTAGCTACTTCTTCTTGG | 31 |  |
| 2 | Enolase | MSH_00070 | eno-1-F | ATGTCAGCAATTAAAAAAATCC | 22 | 202 |
|  |  |  | eno-1-R | CACCTTTTCCACCAAACCAATTAG | 24 |  |
|  |  |  | eno-2-F | CTAATTGGTTTGGTGGAAAAGGTG | 24 | 785 |
|  |  |  | eno-2-R | AAATCCAGCCCAGTCGCTTT | 19 |  |
|  |  |  | eno-3-F | AAAGCGACTGGGCTGGATTT | 19 | 413 |
|  |  |  | eno-3-R | TTTTTTAAGATTGTAAAATGCGTC | 24 |  |
| 3 | Elongation factor Tu | MSH_03475 | ef-tu-F | ATGGCAAAATTAGATTTTGAC | 21 | 1182 |
|  |  |  | ef-tu-R | TTTAACGATTTTTGTAACTG | 20 |  |
| 4 | Hemagglutinin | MSH_01355 | mspb-1F | CAAACTCCAGCACCTGCTC | 19 | 827 |
|  |  |  | mspb-1R | GATTGAGCTGTCCATTTGAATGCTGG | 26 |  |
|  |  |  | mspb-2F | CCAGCATTCAAATGGACAGCTCAATC | 26 | 108 |
|  |  |  | mspb-2R | TTTGAATTCTGATTTTTCTCTAGCTTTGGTCCATGCTCTAGGG | 43 |  |
| 5 | Uncharacterized protein | MSH_01690 | lp78-1-F | GGAGATAAAACTGATGGTGGATCTG | 25 | 99 |
|  |  |  | lp78-1-R | GTTATTAATTCTTTTCCATTCTTCAGGAG | 29 |  |
|  |  |  | lp78-2-F | CTCCTGAAGAATGGAAAAGAATTAATAAC | 29 | 126 |
|  |  |  | lp78-2-R | CTTCTCCTTCGCTCCATGGAGCACCAAG | 28 |  |
|  |  |  | lp78-3-F | CTTGGTGCTCCATGGAGCGAAGGAGAAG | 28 | 552 |
|  |  |  | lp78-3-R | ACCCCACAGTTCTTTAACTGCTTC | 24 |  |
|  |  |  | lp78-4-F | GAAGCAGTTAAAGAACTGTGGGGT | 24 | 482 |
|  |  |  | lp78-4-R | GATCTTCCCCAGTGTGATGTTGAAACA | 26 |  |
|  |  |  | lp78-5-F | TGTTTCAACATCACACTGGGGAAGATC | 26 | 611 |
|  |  |  | lp78-5-R | CATTTTTCCATTTTCCAGGAACA | 22 |  |
|  |  |  | lp78-6-F | TGTTCCTGGAAAATGGAAAAATG | 22 | 129 |
|  |  |  | lp78-6-R | GATAGAAGCCATTTTAGGAATAATCTTGTAG | 30 |  |
|  |  |  | lp78-7-F | CTACAAGATTATTCCTAAAATGGCTTCTATC | 30 | 398 |
|  |  |  | lp78-7-R | GTTGTTAGTAGCTCTAACGGTTGATACAA | 29 |  |
| 6 | NADH oxidase | MSH_02670 | nox-1F | ATGGAAAACAATAAAATTATAG | 22 | 158 |
|  |  |  | nox-1R | CCTCCAACCCAAACAGCAATACC | 23 |  |
|  |  |  | nox-2F | GGTATTGCTGTTTGGGTTGGAGG | 23 | 229 |
|  |  |  | nox-2R | GAACTATAGGCCATGTTCCTCC | 22 |  |
|  |  |  | nox-3F | GGAGGAACATGGCCTATAGTTC | 22 | 869 |
|  |  |  | nox-3R | CTTGTCCCCAAGAACCAAC | 19 |  |
|  |  |  | nox-4F | GTTGGTTCTTGGGGACAAG | 19 | 183 |
|  |  |  | nox-4R | AGCTTTATATTTTAAACCAAGTG | 23 |  |

**Table S2.** Multiple nucleotide sequences alignments of the six genes of *MS* with reference references.

| Country | Strains | Accession number | Nucleotide identities (%) | | | | | |
| --- | --- | --- | --- | --- | --- | --- | --- | --- |
|  |  |  | *dnak* | *enolase* | *ef-tu* | *mspb* | *lp78* | *nadh* |
| America | WVU1853T | NZ_CP011096 | 99.5 | 99.7 | 99.7 | 86.5 | 99 | 99.6 |
| Brazil | 53 | NC_007294 | 99.6 | 99.3 | 99.6 | 90.2 | 99.4 | 99.6 |
| Korea | G3 | CP082195 | 99.9 | 99.5 | 99.7 | 84.5 | 99.4 | 99.9 |
| China | FJ-01 | CP079705 | 99.6 | 99.3 | 99.7 | 87.5 | 99.7 | 99.6 |
| China | HN01 | CP034544 | 99.6 | 99.7 | 99.7 | 87.2 | 98.7 | 99.6 |
| China | SD2 | CP107525 | 99.6 | 99.7 | 100 | 84.8 | 98.7 | 99.6 |
| China | 5-9 | CP083748 | 99.6 | 99.3 | 99.7 | 87.3 | 99.7 | 99.6 |
| China | WF18 | CP069379 | 99.6 | 99.7 | 99.7 | 86.3 | 98.7 | 99.6 |
